# A Functional Portrait of Med7 and the Mediator Complex in *Candida albicans*

**DOI:** 10.1371/journal.pgen.1004770

**Published:** 2014-11-06

**Authors:** Faiza Tebbji, Yaolin Chen, Julien Richard Albert, Kearney T. W. Gunsalus, Carol A. Kumamoto, André Nantel, Adnane Sellam, Malcolm Whiteway

**Affiliations:** 1 Department of Biology, McGill University, Montreal, Quebec, Canada; 2 Department of Biology, Concordia University, Montreal, Quebec, Canada; 3 Department of Medical Genetics, University of British Columbia, Vancouver, British Columbia Canada; 4 Department of Molecular Biology and Microbiology, Tufts University, Boston, Massachusetts, United States of America; 5 Biotechnology Research Institute, National Research Council, Montreal, Quebec, Canada; 6 Department of Microbiology-Infectious Disease and Immunology, Laval University, CHU de Québec Research Center (CHUL), Quebec, Quebec, Canada; University College Dublin, Ireland

## Abstract

Mediator is a multi-subunit protein complex that regulates gene expression in eukaryotes by integrating physiological and developmental signals and transmitting them to the general RNA polymerase II machinery. We examined, in the fungal pathogen *Candida albicans*, a set of conditional alleles of genes encoding Mediator subunits of the head, middle, and tail modules that were found to be essential in the related ascomycete *Saccharomyces cerevisiae*. Intriguingly, while the Med4, 8, 10, 11, 14, 17, 21 and 22 subunits were essential in both fungi, the structurally highly conserved Med7 subunit was apparently non-essential in *C. albicans*. While loss of CaMed7 did not lead to loss of viability under normal growth conditions, it dramatically influenced the pathogen's ability to grow in different carbon sources, to form hyphae and biofilms, and to colonize the gastrointestinal tracts of mice. We used epitope tagging and location profiling of the Med7 subunit to examine the distribution of the DNA sites bound by Mediator during growth in either the yeast or the hyphal form, two distinct morphologies characterized by different transcription profiles. We observed a core set of 200 genes bound by Med7 under both conditions; this core set is expanded moderately during yeast growth, but is expanded considerably during hyphal growth, supporting the idea that Mediator binding correlates with changes in transcriptional activity and that this binding is condition specific. Med7 bound not only in the promoter regions of active genes but also within coding regions and at the 3′ ends of genes. By combining genome-wide location profiling, expression analyses and phenotyping, we have identified different Med7p-influenced regulons including genes related to glycolysis and the Filamentous Growth Regulator family. In the absence of Med7, the ribosomal regulon is de-repressed, suggesting Med7 is involved in central aspects of growth control.

## Author Summary

In this study, we have investigated Mediator function in the human fungal pathogen *C. albicans*. An initial screening of conditionally regulated Mediator subunits showed that the Med7 of *C. albicans* was not essential, in contrast to the situation noted for *S. cerevisiae*. While loss of CaMed7 did not lead to loss of viability under normal growth conditions, it dramatically influenced the pathogen's ability to grow in different carbon sources, to form hyphae and biofilms, and to colonize the gastrointestinal tracts of mice. We used location profiling to determine Mediator binding under yeast and hyphal morphologies characterized by different transcription profiles. We observed a core set of specific and common genes bound by Med7 under both conditions; this specific core set is expanded considerably during hyphal growth, supporting the idea that Mediator binding correlates with changes in transcriptional activity and that this binding is condition specific. Med7 bound not only in the promoter regions of active genes but also of inactive genes and within coding regions and at the 3′ ends of genes. By combining genome-wide location profiling, expression analyses and phenotyping, we have identified different Med7 regulons including genes related to glycolysis and the Filamentous Growth Regulator family.

## Introduction

Mediator is a multi-subunit protein complex that is implicated in the proper expression of most RNA polymerase II (pol II) transcripts, including both protein-coding and non-coding genes [1]–[9]. Mediator, which contains four modules, termed head, middle, tail and kinase, serves as a central scaffold within the pre-initiation complex (PIC) and helps regulate pol II activity in ways that are currently poorly understood. Mediator is generally targeted by sequence-specific, DNA-binding transcription factors (TFs) that work to control gene expression programs in response to developmental or environmental cues [2], [10]–[12]. Overall the Mediator complex thus acts as a bridge, conveying regulatory information from enhancers and other control elements to the basal RNA polymerase II transcription machinery.

Mediator has been found to be required for transcriptional regulation of nearly all RNA polymerase II-dependent genes in *Saccharomyces cerevisiae* [1], [13]–[15] and post-translational modifications of specific Mediator subunits can affect global patterns of gene transcription. The *S. cerevisiae* Mediator complex comprises 21 subunits and it is found both in free form and as a holoenzyme in a complex with pol II [16]–[21]. Mediator structure and function seems to be evolutionarily conserved; the yeast complex contains orthologs of most subunits found in the mammalian complex [22], [23]. However, while the overall modular framework has been conserved, individual Mediator subunit sequences have diverged significantly, such that identity or similarity can be modest between orthologous yeast and human subunits (reviewed in [2], [24]). Additionally, human Mediator contains subunits with no identifiable counterpart in yeast, such as Med13L [25], [26], while the mediator subunits Med3 and Med5 are specific to yeast and have no close relatives in humans or other organisms [27]–[30]. Thus while Mediator has a general structure and function, there is considerable scope for variation in the roles and importance of specific subunits within this overall framework.

Initial efforts to determine functions of the diverse modules of Mediator, proposed that core Mediator (head, middle, tail) and/or holoenzyme might be responsible for Mediator's activities in transcriptional activation, whereas Mediator associated with the kinase module might serve as a transcriptional repressor [1], [31], [32]. This global function of Mediator appears highly conserved among eukaryotes from yeast to humans. In addition to transcriptional activation, core Mediator was found to stimulate basal transcription and support activation of transcription *in vitro* in association with a variety of DNA binding transcription factors, whereas Mediator complexes containing the kinase module did not [33]–[37]. Several other roles of Mediator have been recently proposed; Mediator has been suggested to activate the pre-initiation complex, allow re-initiation during multiple rounds of transcription, post-initiation [1], [3]–[9], [38]–[41], transcription elongation [10], [12], [42], [43], transcription termination [3], [13], [44], [45], chromatin structure regulation [9], [18]–[21], [45], [46], sub-telomeric silencing [4], [5], [23], [47]–[50] and mRNA processing [15], [24], [51], [52]. Additionally, functional analyses of several mediator subunits were studied. For example Med31 and Med20 are needed for filamentous growth and biofilm formation [53], *MED31* deletion shows a cytokinesis defect [53], *MED3* deletion results in a filamentous growth defect [54] and different subunits play a role in white-opaque switching [55].

Several studies support the concept that the mediator modules (head, middle, tail, and kinase) provide both specific and common functions [15], [26]. For example, the *S. cerevisiae* head module bind both Pol II and general initiation factors and can stimulate basal transcription even in the absence of the remaining Mediator subunits; however, the head module alone does not support activator-dependent transcription [27]–[29]. Different subunits from the middle module play very specific roles. The mammalian Med1 C-terminal region includes LXXLL motifs, which bind to multiple nuclear receptors in a ligand-dependent fashion; this interaction is thought to be sufficient to recruit Mediator to many nuclear receptor-regulated genes [31], [47], [48], [50], [56]. Med14 appears to be at the interface of the middle and tail modules and may contribute to the overall organization of Mediator [33], [57]. The *S. cerevisiae* tail module appears to function in recruitment of Mediator to genes through direct interactions with various DNA binding trans-activators [38]–[41], [58]. In addition to transactivation, there is evidence that the kinase module can act as a repressor of Pol II mediated transcription, so overall the mediator complex appears to be able to play many roles [42], [43].

The Mediator complex is big enough to offer a surface for interactions with numerous transcription regulators. In addition to activator interactions, Mediator also interacts with various cofactors (co-activators and co-repressors) grouped into the chromatin modifying factors (histone-modifying and ATP-dependent nucleosome remodeling enzymes) and general cofactors (TFIID and the Mediator complex itself) [3], [44], [45]. Since Mediator can make diverse protein–protein interactions that may differ at distinct promoters, and can furthermore adopt activator dependent conformations, it has been viewed as a potential modulator or integrative hub capable of generating diverse outputs from physiological signals in a promoter-specific fashion [9], [46]. In addition to all these roles, several studies have served to define the specificity of this complex. It has been shown that different activators can employ one or more distinct activator binding regions of Mediator subunits to achieve specificity [4], [5], [47]–[50]. Specific mediator subunits are required to bind to certain promoter sequences [15], [51], [52]. Mediator can be a condition-specific [15], [51], [52] and species-specific inducer of activation [53]. Additional complexity in Mediator function is introduced by the tissue-specific role of individual Mediator subunits in higher organisms [30], [32].

The three dimensional structure of the Mediator complex reflects both these general and specific characteristics [57], [59]. The head, acting as the universal module of the complex, is responsible for the proper orienting of pol II onto the promoter sequence in order to form the initiation complex. The tail is believed to contribute to the specificity of Mediator, as its subunits bind to specific promoters and/or specific transcription factors. A combination of biochemistry, X-ray crystallography, yeast phenotyping, and transcriptome analysis established a sub-module formed by the N terminal of the *S. cerevisiae* Med7 (Med7N) and Med31 in the mediator middle module; this sub-module is structurally and functionally distinct and is required for activated transcription [58]. It appears that Med7N forms a non-essential tether for the compact peripheral Med31 subunit and is flexibly linked to Med7 C terminal region (Med7C), which forms an extended essential heterodimer with Med21. Overall it appears metabolic sensing, stress response, and certain amino-acid biosynthesis pathways are generally affected by deletion of different Mediator sub-modules, including Med7N.

Here we have investigated Mediator function in the human fungal pathogen *C. albicans*. An initial screening of conditionally regulated Mediator subunits showed that the Med7 of *C. albicans* was not essential, in contrast to the situation noted for *S. cerevisiae*. Because a recent comparison of the Mediators of the *C. albicans* and *S. cerevisiae* also identified that the single *MED2* gene of the budding yeast was represented by the multi-gene TLO family in the pathogen [54], it is evident that there are functionally relevant distinctions between the Mediators of the two ascomycetes. We used location profiling to determine Mediator binding under distinct conditions, and established the functional consequences of loss of Med7 in the pathogen. Although not required for viability, loss of Med7 impacts many aspects of *C. albicans* cell function, and compromises the pathogen's capacity to colonize a mammalian host.

## Results

### Analysis of Mediator subunit mutants in the GRACE collection

**Figure 1:**
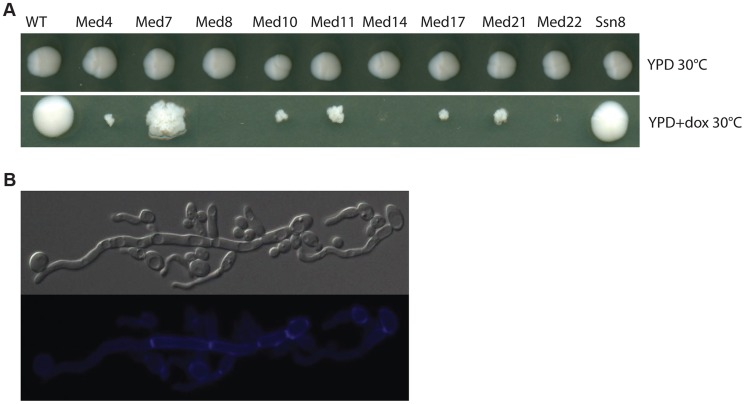
Growth of Mediator subunit mutant strains in the GRACE collection. Cultures of wild type C. *albicans*, and the mediator subunit mutant strains from the Grace collection were grown overnight at 30°C. (**A**). Strains were serially diluted and grown on plates under non-repressing (YPD) or standard repressing conditions (YPD+20 µg/ml doxycycline) for 2–3 days at 30°C and the resulting colonies photographed. Cells from wrinkled colonies were observed by microscopy using DIC for bright field or through the DAPI filter for calcofluor white staining. (**B**). Strains lacking Med7 grow primarily as pseudohyphal cells, as judged by staining with calcofluor white.

A functional genomics approach based on the GRACE (gene replacement and conditional expression) strain collection [60] was used to test the essentiality of mediator subunits. We identified in this collection the genes encoding subunits of each of the complex sub-regions (Tail: *MED14/RGR1*; Middle: *MED4, MED7, MED10/NUT2, MED21/SRB7*; Head: *MED22/SRB6, MED11, MED17/SRB4, MED8*; and Kinase: *SSN8*). Strains were serially diluted and grown on plates under non-repressing (YPD) or standard repressing conditions (YPD+100 µg/ml tetracycline or 20 µg/ml doxycycline) for 2–3 days at 30°C and the resulting colonies were photographed (Figure 1). The strains analyzed fell into three classes; (1) tetracycline-induced repression of the genes for Med8, Med14 and Med22 dramatically inhibited growth at almost all cell concentrations examined, (2) repression of the genes for Med4, Med10, Med11, Med17 and Med21 blocked growth, but this growth inhibition was only easy to see at lower cell concentrations, (3) the repression of the genes for Med7 and Ssn8 did not dramatically reduce growth, although the colony morphology of the *MED7* deletion strain was highly wrinkled and the cells show a pseudohyphal form (Figure 1A and 1B).

We also tested the invasiveness of all the Mediator-subunit Grace strains. Invasive growth – penetration into the agar surface by pseudohyphae – was scored by examination of the colony perimeter and by viewing cell retention after washing the colony from the agar surface. All Mediator complex core subunit mutants tested (*med4*, *med7*, *med10*, *med11*, *med14*, *med17*, *med21*, and *med22*) showed strongly enhanced invasiveness and produced very dense pseudohyphal cells; the growth of the *med8* mutant strain was so poor under these conditions that invasive growth was impossible to score (Figure S1). However, under hyphal induction conditions (serum at 37°C, M199 at 37°C and Spider media at 37°C), when tetracycline was added to inhibit gene expression the majority of the Mediator-complex-subunit GRACE mutants showed a reduction in the wrinkled phenotype or in peripheral hyphal growth. Overall the depletion of the non-essential *SSN8* did not dramatically impact colony morphology, while repressing the *MED7* gene caused an apparent decrease in hyphal characteristics at the colony morphology level (Figure S2).

Most of the Mediator-subunit-mutants in the GRACE collection were derived from genes that were identified as essential for growth in large-scale surveys in the model yeast *S. cerevisiae*, with the exception of the kinase subunit Ssn8. Therefore the robust growth of the *MED7* shut-off strain was surprising since sequence similarity alignments suggest that the Med7 is the most highly conserved subunit of the fungal Mediators. The essential nature of GRACE strains can be independently monitored by testing for cell growth in the presence of 5′-FOA, so we examined the apparent non-essentiality of the genes for Mediator subunits Med7 and Ssn8 in *C. albicans* by testing for growth on 5′-FOA-containing medium. In this assay, the *MED7* and *SSN8* mutants gave frequent 5′-FOA resistant colonies (Figure S5), further suggesting that neither subunit was essential for *C. albicans* growth.

We next tested the non-essentiality of *MED7* by generating a complete knock out of both alleles of the gene through standard disruption approaches. One allele was replaced with the *LEU2* selection marker and the second allele was replaced by *HIS1*. The total removal of the functional *MED7* gene and its replacement with the selection markers was confirmed by PCR (Figure S3). Thus the Med7 subunit, although highly conserved structurally among the eukaryotes and essential for viability in the model yeast *S. cerevisiae*, is not essential in *C. albicans*, and we have investigated the characteristics of CaMed7 in more detail.

### Med7 physical interaction

Because the essentiality of *MED7* was different between *C. albicans* and *S. cerevisiae*, we determined the protein-protein interactions of CaMed7 to confirm it is a *bona fide* subunit of the Mediator complex. We performed a classic TAP purification procedure using a TAP-tagged CaMed7 with an untagged control to remove all non-specific interacting proteins. Mass spectrometric analysis of in-gel digestion of four Gelcode blue stained SDS-PAGE bands of purified proteins after the IP procedure identified 141 *C. albicans* proteins and a further TCA-precipitation led to the identification of an additional 38 proteins (Table S2). In total, 179 *C. albicans* proteins were identified; these included 15 of the 25 *C. albicans* mediator complex subunits, confirming the association of CaMed7/C3_02440C_A with the Mediator complex. We compared the coverage generated through our experiment to that of the orthologous Med7 protein-protein interactions in *S. cerevisiae* available from the Biogrid database (http://thebiogrid.org/34241/summary/saccharomyces-cerevisiae/med7.html) [61]. Interestingly, while the core mediator has been found to be bound by Med7 in the same manner in the two organisms (Med15, Med11, Med4, Med6, Med7, Med8, Med10, Med14, Med16, Med20, Med17, Med18, Med21) the rest of Med7 protein interactions detected in the two species were quite different.

### Expression profiling of CaMed7

Because *C. albicans* cells that had lost Med7 function were still viable, we investigated whether its loss will impact overall gene expression. We used the *Med7* tetracycline-repressible mutant to determine the transcriptional consequence of Med7 depletion by tetracycline; we measured the transcriptional differences between Med7 cells growing in YPD at 30°C in the presence and absence of tetracycline using whole-genome microarrays. Using a statistical-significance analysis with an estimated false-discovery rate of 5%, in addition to a cutoff of 1.5-fold, we identified 140 genes that require Med7p for their proper expression; 56 genes were up-regulated and 84 were down-regulated (Table S3 in the supplemental material). Among these down-regulated genes were genes involved in glucose transport (*HGT8* and *HGT7*), galactose catabolism (*GAL7* and *GAL10*), pH response (*RIM101*, *GLT1*, *CCP1* and *FRE7*), and cell-cell adhesion (*ALS2* and *ALS4*). Among the 56 up-regulated genes were genes involved in iron assimilation (*CFL2*, *CFL4*, *FTR1*, *FRE10*, and *CCC2*) and the hyphal cell wall (*HWP1, RBT1, CSA1, HYR1, SUN41*). Therefore, while the shut-off of Med7 does impact transcription, the number of genes whose expression is modified is modest, and the primarily affected cellular processes are not essential.

**Figure 2:**
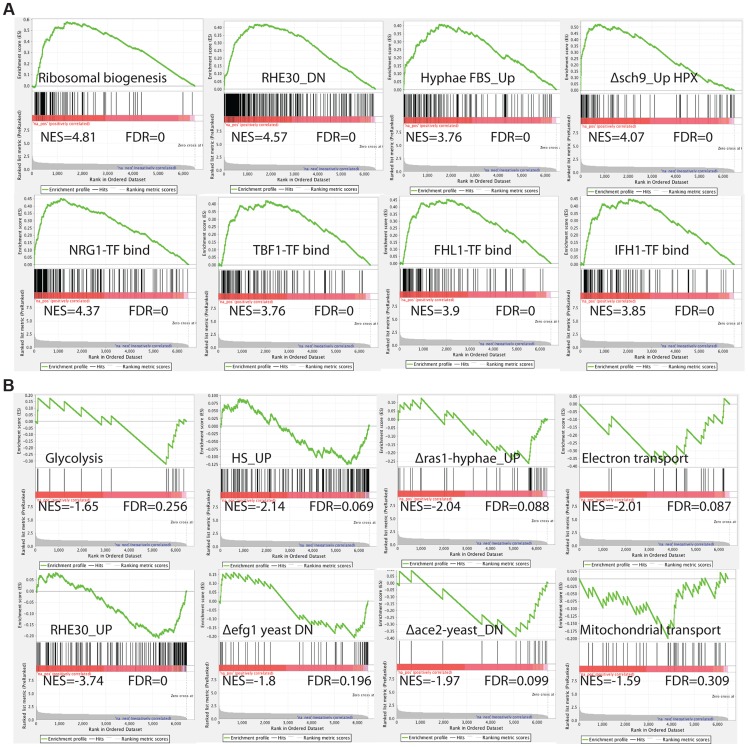
GSEA analysis of the genes differentially expressed in the absence of Med7 in *C. albicans*. Example GSEA enrichment plots for selected genes sets differentially expressed in the *med7* GRACE mutant grown in yeast cell conditions is presented. On the x-axis are genes ranked according to their expression in the *med7* GRACE mutant, from the up-regulated genes on the left hand side to the down-regulated genes on far right. Black vertical lines show the positions of the individual genes in the gene set. The cumulative value of the enrichment score (y-axis) is represented by the green line. A positive normalized enrichment score (NES) indicates enrichment in the up-regulated group of genes in the *med7* GRACE mutant (A), while a negative NES indicates prevalence of the genes in the down-regulated group (B). The title for each of the graphs indicates the genes set used to compare to the *med7* GRACE mutant set. FDR is the false discovery rate. The full Correlation is presented in the Table S5.

To comprehensively investigate the *C. albicans* cellular pathways whose expression is influenced by inactivation of by Med7, we performed Gene Set Enrichment Analysis (GSEA) [62] (Figure 2 and Table S5). GSEA compares (see http://www.broadinstitute.org/gsea/ for details), to a predefined gene set (a custom database of 8123 gene sets (http://www.candidagenome.org/download/community/GSEA_Nantel_2012/) constructed using GO annotations and protein interaction data from CGD (PMID: 19808938), SGD (http://www.yeastgenome.org) and BioGRID [61], most currently published *C. albicans* transcriptional profiling and ChIP-CHIP experiments, our own TF motif database (PMID: 18342603), and *S. cerevisiae* genetic-association data (PMID: 20093466)), a list from the transcript profile of interest created by ranking all of the genes according to the change in their expression, and then asks if a specific gene set is enriched in the top (up-regulated genes) or the bottom (down-regulated genes) of the ranked list. Within the set of genes up-regulated in the *med7* mutant, GSEA detected enrichment for rRNA and ribosome biogenesis; genes important for virulence-promoting functions in *C. albicans*, including genes down-regulated during reconstituted human oral epithelial cells [63] as well as genes differentially expressed in conditions which alter cellular morphogenesis, such as the induction of hyphal growth [64], [65], and the up-regulated transcripts from the *sch9* mutant grown under hypoxia [66]. The GSEA analysis also shows a correlation with Nrg1, Tbf1, Fhl1 and Ifh1 transcription factor binding [67]–[70], as well as translation and genes repressed by the rapamycin; suggesting that the regulation of the ribosomal protein regulon has been compromised.

In the set of down-regulated genes in the *med7* mutant, the GSEA showed enrichment of genes required for electron transport, mitochondrial transport, glycolysis and carbohydrate kinase activity functions. Enrichment was also found in gene sets important for virulence-promoting functions in *C. albicans*. Those include genes up regulated in reconstituted human oral epithelial cells [63] as well as genes differentially expressed in conditions which change morphogenesis, such as hyphal growth regulation (down-regulated genes in yeast *ace2* and yeast *efg1* mutants and genes up-regulated in response to heat shock).

Because of the wrinkled colony morphology and the evidence for changes in the expression of gene sets implicated in the hyphal transition, we also investigated the impact of the tet-repressed allele of *MED7* on gene expression under hyphal growth conditions. We performed a Med7 conditional mutant expression profiling under the hyphal inducing conditions of 37°C in the presence of 10% serum. Using a statistical-significance analysis with an estimated false-discovery rate of 5%, in addition to a cutoff of 1.5-fold, we identified 521 genes that require Med7p for their proper expression, including 261 up-regulated genes and 260 down-regulated genes (see Table S4 in the supplemental material). This set of genes was much larger than that observed during yeast growth, suggesting a potentially more significant role of Med7 in hyphal growth. To explore the biological processes controlled by the Med7 during growth in hyphae-inducing conditions, we conducted a gene ontology (GO) investigation by analyzing the up- and down-regulated genes. The up-regulated transcripts were enriched in ribosome biogenesis, RNA metabolic processes, translation, and transport functions while the repressed transcripts were enriched in regulation of cell growth, invasive growth in response to glucose limitation, invasive filamentous growth and growth of unicellular organism as a thread of attached cells (Table S4). To reveal the cellular pathways regulated by Med7 in the hyphal induced condition, we also performed GSEA analysis (Table S5). Similar to the yeast growth condition data, in the set of genes up-regulated in the *med7* mutant in the presence of serum at 37°C, GSEA detected enrichment for rRNA and ribosome biogenesis; translation, nucleolus cell function, as well as correlations with Nrg1, Tbf1, Fhl1, and Ifh1 TF binding, translation and genes repressed by rapamycin. In the down-regulated gene set, GSEA uncovers similarity with cell cycle genes, regulation of metabolism, regulation of transcription, sugar transport, chromatin modification and gene set repressed in *rim101* and *nrg1* mutants. Based on GSEA analysis, the depletion of Med7 in both yeast and hyphae conditions shows a strong correlation with functions related to the regulation of ribosome biogenesis in *C. albicans*, including binding of the Tbf1, Fhl1, and Ifh1 TFs, translation, and genes repressed by rapamycin, which would implicate a possible role of Med7 in growth control by communicating nutriment status to ribosome biogenesis rate.

While transcription profiling of the GRACE strain mutant under induced and repressed conditions provided an overview of the cellular functions influenced by the Med7 subunit, many transcriptional effects could be indirect consequences of modifications in the function of transcription factors. Therefore we decided to couple these transcriptional profiling results with location profiling to identify Med7-binding sites.

### Genome-wide mapping of the Med7 subunit

We determined the genomic occupancy of Med7 using chromatin immunoprecipitation coupled with microarray analysis (ChIP-Chip). ChIP was performed on cells grown to mid-logarithmic phase in rich medium. Input and immunoprecipitated samples were amplified and binding locations were examined in duplicate ChIP-chip experiments using high density tiling arrays [71], [72]; the binding pattern was determined for cells grown in both yeast and hyphal conditions. Signal intensities of the tiling array were normalized using a spatially centered LOESS scatterplot smoothing scheme (using an in-house software implementation). Using a log ratio = 0.4 as a cutoff, we identified 318 open reading frames (ORFs) that have a binding site in the 1500 bp 5′ region in the yeast growth condition and 812 targets in the hyphal growth condition (Table S6). In general the binding was in the intergenic regions and upstream activating sequences. However we also observed binding in the middle of ORF and at the 3′ends of genes; this general pattern was also seen for other Mediator subunits in *S. cerevisiae* and *S. pombe* [73]–[75].

We detected around 600 peaks in the yeast growth condition and 1400 peaks in the hyphae-inducing condition (Table S7). These represent nearly 10 and 30%, respectively, of all *C. albicans* genes; this occupancy correlates well with the Med7 binding % in *S. pombe* [75] and *S. cerevisiae* [73]. Overall the comparison between the targets bound by Med7 under yeast and hyphal growth conditions identified a core of 200 genes bound under both conditions (Table S6). This core represented the bulk of the yeast-form-bound genes; only about 100 genes were identified as yeast specific. By contrast, the hyphal growth condition expanded the core binding set extensively, with more than 600 genes characteristic of the hyphal condition (Table S6). Overall, this result supports the idea that Med7 binding is condition specific.

**Figure 3:**
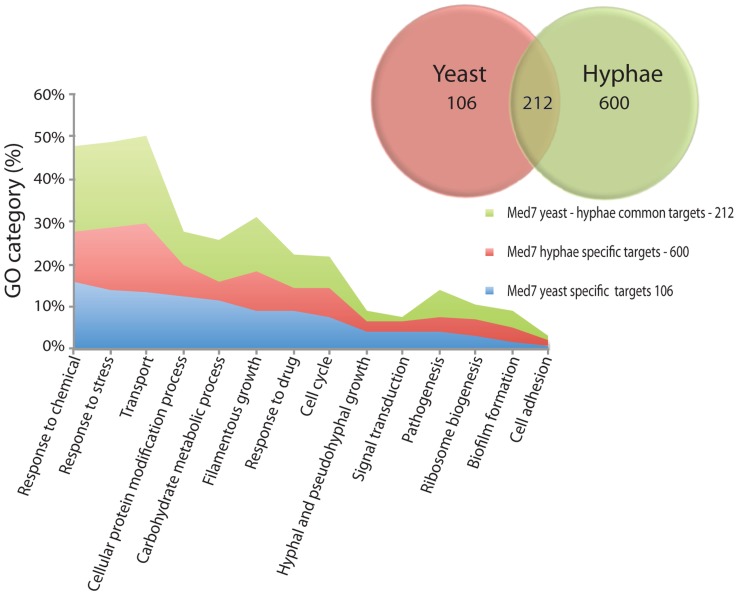
GO enrichment of yeast and hyphae binding targets for Med7p. Targets with a log ratio = 0.4 enrichment and binding site in the 1500 bp 5′ region of the ORF (318 and 812 genes for Med7 in yeast and hyphae conditions respectively) were analyzed with the CGD GO Term Finder (http://www.candidagenome.org/cgi-bin/GO/goTermFinder). The *p*-value of enrichment for each GO category is indicated. Overlap number of yeast and hyphae binding targets are represented. Genes associated with transport, response to stress, carbohydrate metabolic processes, filamentous growth, and cellular protein modification processes, response to drugs, cell cycle and pathogenesis were noticeably enriched.

A comparison between the yeast and hyphal growth data suggests that Med7 promoter occupancy correlates with high transcriptional activity when mapped against our previous data [72]. In yeast growth conditions, from the 318-med7 targets in the combined data set only 139 were active (Table S8). To assess the biological processes controlled by the Med7p, we conducted a gene ontology (GO) investigation analyzing all genes whose promoters are associated with Med7p (Figure 3). Genes associated with transport, response to stress, carbohydrate metabolic processes, filamentous growth, and cellular protein modification processes, response to drugs, cell cycle and pathogenesis were significantly enriched in the yeast dataset (Table S6). Examination of overall Med7p binding targets revealed that they were enriched in the promoters of genes coding for TFs and general transcriptional regulators including the key repressors Nrg1p; Ssn6p, Efg1 and Mig1p that control genes involved in sugar metabolism.

In the hyphal condition, no enrichment of direct binding to hyphal specific genes, such as *HWP1*, *ESE1*, *MSS11*, *PGA7* or *TEC1*, was found. However, Med7 binds a large number of the *FGR* (filamentous growth regulator) family (*FGR3; FGR42; FGR50; FGR51; FGR6-1; FGR6-10; FGR6-3; FGR6-4; GPI19*), as well as cell adhesion factors (*ALS1*, *TDH3* and *DEF1*) and regulators such as *ACE2*. Med7p targets were notably enriched in other functional categories such as stress response, carbohydrate metabolism, transport, pathogenesis and the cell cycle (Figure 3 and Table S6). This suggests a promoter-specific function of Mediator.

By cross-referencing the genome-wide location data with the list of Med7p transcriptionally controlled genes, we were able to identify genes whose transcription, during the yeast and/or hyphal growth phases, is influenced by the loss of Med7. In yeast and hyphal conditions, a total of 19 and 83 common genes, respectively, were common between the Med7 ChIP-Chip data and genes that require Med7 for their proper regulation (Table 1). From 19 Med7p direct targets during yeast growth only two genes were activated, whereas 17 were repressed in the *med7* mutant. The 83 common targets between expression profiling and the binding list in the hyphal condition contain 37 up-regulated and 46 down-regulated genes (Table 1). All together, although Med7 changed its essentiality between *S. cerevisiae* and *C. albicans* it is still has a role in transcriptional regulation through condition specific binding in the pathogen. To better understand Med7 function in *C. albicans*, we looked at different cellular phenotypes of the *med7* deletion mutant.

**Table 1:** Common genes between Med7 Binding target and Med7 expression profile.

	Med7 up genes in expression profile found in ChIP-Chip data	Med7 down genes in expression profile found in ChIP-Chip data
**Yeast**	2 genes	17 genes
	TPI1	ZCF2
	PGA32	TPS2
		GAL10
		MAF1
		TPO3
		HSP21
		LEU42
		GLK4
		GAL7
		PDX3
		OSM1
		ADH5
		orf19.3352
		orf19.4617
		orf19.6491
		orf19.4818
		orf19.7566
**Hyphal**	37 genes	46 genes
	ZRT2	AVT4
	YTM1	SUT1
	TPS1	TPO3
	SIM1	UBC4
	RPS7A	YHB1
	RPS3	PHO4
	RPS21	PMC1
	RPS10	RPN4
	RPS1	RSN1
	RPL7	SUI1
	RPL35	HGT6
	PST2	HOS3
	PGA45	HSK3
	PGA32	IPK1
	RPL39	TCO89
	PGA17	MET4
	NHP2	PCL5
	MCR1	FRP5
	GLY1	FUN31
	GCV3	GAL10
	GCR3	GPR1
	FDH3	GPX2
	CHO1	HGT1
	ATP3	ALS1
	AKR1	ARA1
	AAT1	BRG1
	orf19.7478	CDC21
	orf19.715	CRP1
	orf19.6701	FCR3
	orf19.5614	orf19.6897
	orf19.527	orf19.7227
	orf19.4503	orf19.725
	orf19.3924	orf19.7250
	orf19.3481	orf19.449
	orf19.2457	orf19.6391
	orf19.1890	orf19.6789
	orf19.1709	orf19.449
		orf19.4643
		orf19.4888
		orf19.5813
		orf19.215
		orf19.2547
		orf19.3473
		orf19.3712
		orf19.1286
		orf19.2049

### A phenotypic profile of the *Candida albicans* Med7 subunit

To investigate the *CaMed7* homozygote null mutant phenotype, we first compared the null mutant with the tet-repressed *Med7* Grace mutant. Somewhat surprisingly, the *Med7* null mutant generated normal looking smooth colonies when cultured under yeast growth conditions, in contrast to the wrinkled colonies generated by repression of the Grace Med7 strain. To exclude the possible effect of tetracycline, we also grew the *med7* null mutant on YPD media supplemented with the tetracycline, but saw no effect on the phenotype, showing that the tet-repressed and true null colony phenotypes were not identical. This could result from colony morphology being differentially affected by a sudden loss of function in the repressed cells in contrast to the sustained and permanent loss of function exhibited by the double disruption mutant. Alternatively, because Med7 itself is part of the transcriptional machinery, full tetracycline-repressed inactivation may be complicated because as Med7 function is reduced, the ability to repress transcription may itself be affected. Although both approaches to test gene essentiality show that the *MED7* gene is not essential, it appears that the two strategies for gene inactivation do not produce strains that are physiologically identical; in our subsequent phenotypic analyses we focused on the characteristics of the true null mutant.

**Figure 4:**
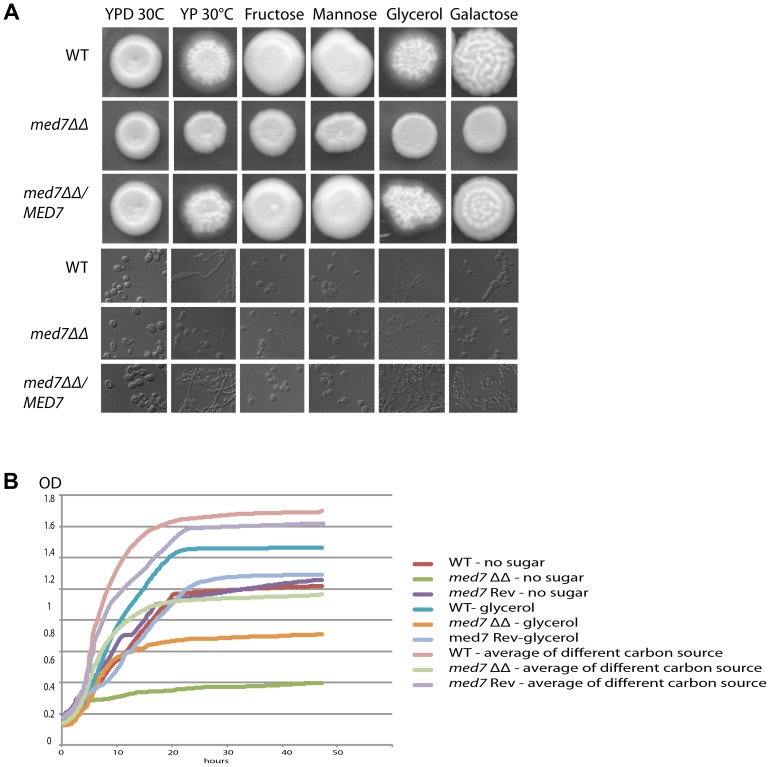
Med7 is required for carbohydrate metabolism of *C. albicans*. Phenotypic assay of *Med7* into diverse carbon sources in agar and liquid condition at 30°C. (A) *med7ΔΔ*, *med7* revertant and WT Cells were serially diluted and a representative dilution is displayed on YP+different sugar source at 2% at 30°C. Pictures were taken after 2–3 days of growth. WT refers to strain *SN148*. (B) Growth assay in liquid medium with the different carbon sources and without any carbon source. OD_595_ readings were taken every 10 min during 48 h at 30°C using a Tecan –Sunarise reader. The graphics show an average of different carbon source growth curves (Figure 4S shows the graphics for each carbon sources).

**Figure 5:**
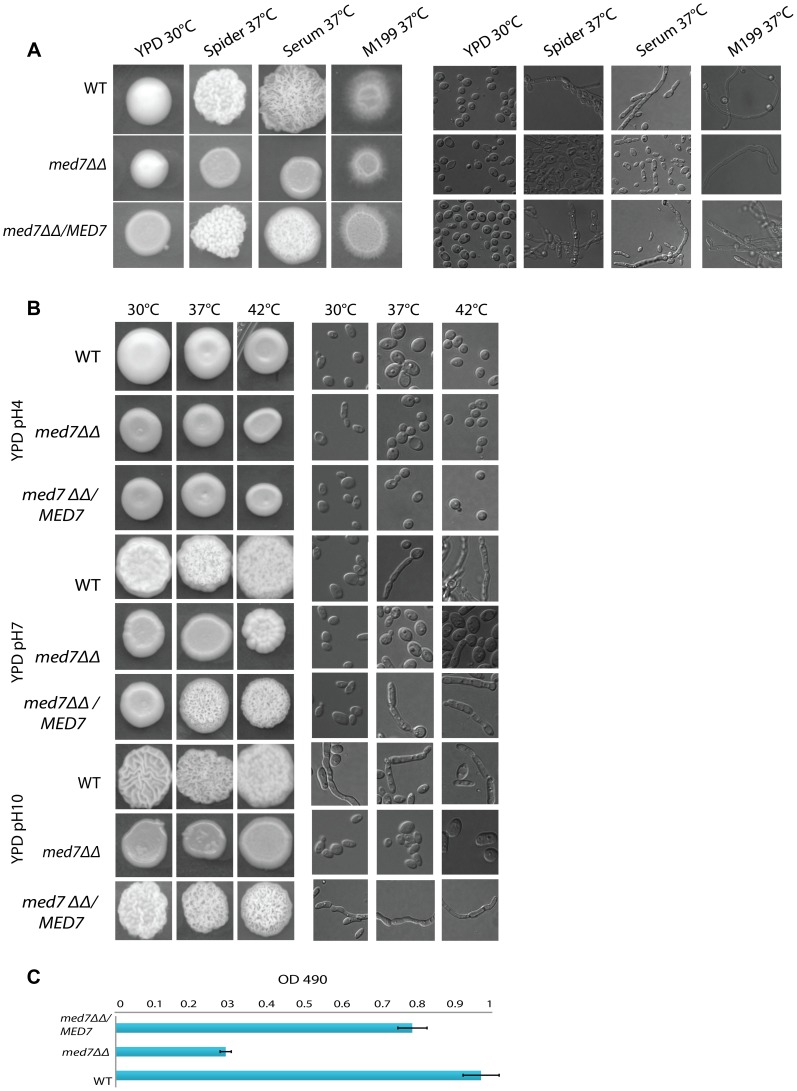
Med7 is required for hyphal development in *C. albicans*. Phenotypic assay of Med7 including morphology using yeast and hyphae promoting conditions-YPD 30°C, Spider, Serum, and M199 at 37°C (**A**) and stress condition temperature, and pH (**B**) and biofilm assay (**C**). (**A**) Wild type, Med7 complement or *med7* mutant cells were serially diluted and were displayed on plates containing hyphae-inducing media and incubated at 37°C for 3 days. The colonies were photographed using a Scanner. Deletion of *MED7* blocked (in YPD+10%FBS, and Spider at 37°C) or reduced (in M199 media at 37°C) filamentous growth. (**B**) Wild type, *med7* mutant and revertant cells were serially diluted and were displayed on YPD plates at pH 4, 7 and 10 and incubated at 30°C, 37°C and 42°C for 3 days. The colonies were photographed using a Scanner. The *med7* mutant was unable to undergo filamentous differentiation compared to the wild type and complementary strains. (**C**) Biofilms were grown in standard C. albicans RPMI growth conditions, and metabolic activity was quantified. Error bars indicate SEM. Each of the above growth assays was performed in six replicates for each of the mutant, revertant and WT. The homozygous diploid *med7* deletion mutant shows a 3-fold decrease in biofilm formation compared to WT.

Phenotypic profiles for *med7*ΔΔ were established by investigating a set of different conditions. We tested the phenotypic consequences of growth on different carbon sources, under different temperatures, pHs and stress conditions, and we examined the effect of signals that induce morphological changes. The assay conditions have been separated into the broad categories of nutrition (Figure 4: YPD and different carbon sources), morphology (Figure 5A: YPD 30°C, Spider, Serum, and M199 at 37°C), and stress (Figure 5B: temperature, pH).

We tested the ability of the *med7*ΔΔ strain to grow on the fermentable carbon sources glucose, fructose, mannose, and galactose, the non-fermentable carbon source glycerol, and on YP medium without any carbon source. On solid YP dextrose, fructose and mannose media, both the *med7* deletion and the WT strains formed smooth colonies, while on glycerol, galactose and sugarless medium the WT and revertant strains formed colonies with filamentous peripheries (Figure 4A). In all cases the *med7ΔΔ* strain grew somewhat more slowly than the WT strain judged by the size of the colony. Because the agar provides poorly defined carbon substrates capable of supporting *C. albicans* growth [76], we also tested growth in liquid medium with the different carbon sources, as well as with SC not supplemented with any carbon source (Figure 4B and Figure S4). While the mutant and WT strains grew with similar kinetics in the YP glucose liquid medium, the mutant strain was slower growing in the galactose, fructose, mannose and glycerol media, and failed to grow detectably in the SC medium lacking a carbon supplement. Genes associated with carbohydrate metabolic processes were significantly enriched in our Med7 GRACE mutant expression profile dataset and our Med7 mapping by ChIP-Chip (Table S3 and S6).

We also screened the *med7ΔΔ* strain under different stress conditions including temperature (30°C, 37°C and 42°C), and pH (4, 7 and 10). Figure 5B shows the different phenotypes obtained. At 30°C the *med7* mutant did not show any growth defect at the acid and neutral pH conditions tested: however, when grown at alkaline pH, the Med7 mutant did not make hyphae while the WT strain formed highly filamentous colonies. At 37°C the *med7* mutant had a filamentation growth defect in alkaline and neutral pH compared to the WT, however we could not see any difference in acidic media. The same phenotype seen at 37°C was found at the higher temperature of 42°C. In response to numerous environmental cues, deletion of Med7 blocked (in YPD+10%FBS, and Spider at 37C) or reduced (in M199 media at 37C) filamentous growth (Fig. 5A). Genomic occupancy of Med7 under hyphae-promoting condition showed bindings to large number of *FGR* (filamentous growth regulator) family members (*FGR3; FGR42; FGR50; FGR51; FGR6-1; FGR6-10; FGR6-3; FGR6-4; GPI19*), in addition to genes required for cell adhesion such as *ACE2*, *ALS1*, *TDH3* and *DEF1*. The *C. albicans* FGR genes were identified in a haploinsufficiency screen [77] and did not have close relatives in *S. cerevisiae* or other model organisms. Because they lacked close relatives in *S. cerevisiae*, it was suggested that these genes could be implicated in aspects of filamentous growth that are specific to the ability of *C. albicans* to colonize and proliferate in warm-blooded animals, the typical hosts for this fungus.

The *Med7* mutant was further tested for biofilm formation on a plastic surface performed in six replicates for each of the mutant and WT strains as described in the Materials and Methods section. The homozygous diploid *med7* deletion mutant showed a 3-fold decrease in biofilm formation as compared to the WT (Fig. 5C), suggesting that Med*7* plays a role in biofilm development.

### The Med7 subunit is implicated in murine gastrointestinal colonization by *C. albicans*

**Figure 6:**
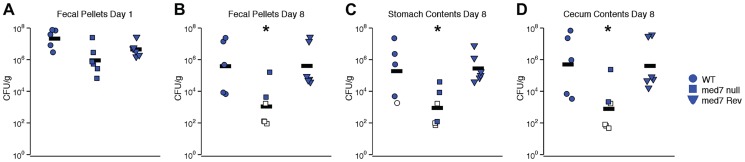
*C. albicans med7* null mutant strain has a colonization defect compared to WT or *med7* revertant strains. Cells of strain SC5314 (WT; circles), *med7* null (squares) or *med7* revertant (triangles) were inoculated into female Swiss Webster mice by oral gavage (5×10^7^ CFU/mouse). Colonization was measured in fresh fecal pellets on days 1 (**A**) and 8 (**B**) post-inoculation and in stomach (**C**) and cecum (**D**) contents harvested on day 8 post-inoculation. *C. albicans* CFU/g of material was determined. Each symbol represents a sample from an individual mouse; bars indicate geometric means. Open symbols indicate that no colonization was detected, and CFU/g was assumed to be equal to the limit of detection; data from two experiments is shown. Asterisks indicate that *med7* null colonization is lower than either WT or *med7* Rev: *, p≤0.05 (pairwise t-test, Bonferroni correction).

Defects in filamentation, biofilm formation and carbohydrate utilization are likely to have profound effects on the ability of *C. albicans* cells to function in the natural environment, in particular to function as a commensal of a mammalian host. To determine whether gastrointestinal colonization by *C. albicans* is influenced by loss of *MED7*, we orally inoculated antibiotic-treated Swiss Webster mice with WT, *med7* null mutant and *MED7* reconstituted null mutant *C. albicans* (5×10^7^ CFU/mouse). Each strain shown here was evaluated in 5 (WT) or 6 (*med7* null mutant and *MED7* reconstituted null mutant) mice, in two independent biological experiments. Intestinal tract colonization was measured in fresh fecal pellets on days 1 (Fig. 6**A**) and 8 (Fig. 6**B**) post-inoculation and in stomach (Fig. 6 **C**) and cecum (Fig. 6 **D**) contents harvested on day 8 post-inoculation. On day 1 post-inoculation, the *med7* null mutant colonization was significantly lower than the WT, but not the *MED7* heterozygous strain (Fig. 6A). On day 8 post-inoculation, *med7* null colonization was significantly lower (p≤0.05 pairwise t-test, Bonferroni correction) than either WT or the revertant in fecal pellets, stomach contents and cecum contents.

## Discussion

### Med7 gene essentiality in *C. albicans*

Mediator is an evolutionarily conserved protein complex that connects transcription regulators to the RNA polymerase complex in eukaryotic cells. Mass spectrometry approaches and the availability of a complete list of mammalian Mediator subunits, together with the sequencing of genomes from a number of species, made it possible to determine the evolutionary relationships among subunits of *S. cerevisiae* and higher eukaryotic Mediators. To date, orthologs of a minimum 22 out of 25 *S. cerevisiae* subunits have been identified in higher eukaryotes, and Mediator complexes from all organisms appear to have a similar, evolutionarily ancient, modular organization. However, although the overall structure is maintained, some of the subunits appear to have significantly changed their roles between organisms. For example, Med15, which is not essential in *S. cerevisiae*, is observed to be required for viability in *S. pombe* [78] and the *MED2* gene of *S. cerevisiae* has been replaced by a family of *TLO* genes in *C. albicans* [54].

We have investigated several aspects of the Mediator complex of the opportunistic pathogen *C. albicans*. Screening a variety of genes encoding Mediator subunits that function in each of the subdomains of the complex showed that, in general, subunits that were essential for the viability of the yeast *S. cerevisiae* were also essential for the viability of the distantly related ascomycete *C. albicans*. Somewhat surprisingly, the highly structurally conserved Med7 subunit was not essential for viability in the pathogen, although it was essential in *S. cerevisiae*. We epitope tagged this non-essential subunit to map chromatin association sites, and analyzed *C. albicans* strains with an inactivated Med7 subunit to determine the phenotypic consequences of the loss of Med7 function on global properties such as gene expression and host colonization, as well as more specific phenotypes such as filamentation and carbon source utilization that were predicted to be impacted through the analysis of the transcriptional consequences of the mutation. Overall, although Med7 was not essential for *C. albicans* viability, its loss had profound consequences on a variety of cellular functions, and ultimately resulted in a strain that was severely compromised in host colonization.

### Med7 has a role in carbon metabolism regulation and host colonization

This analysis of the Med7 role in host colonization focused on the mammalian GI tract. Oral inoculation of antibiotic-treated Swiss Webster mice with WT, *med7* null mutant and *MED7* reconstituted revertant strains showed that Med7 plays a significant role in the ability of the cells to colonize; cells lacking *MED7* failed to establish within the GI tract (Figure 6). Previous analysis of transcriptional regulatory circuits and the repertoire of genes that *C. albicans* uses to exploit niches within its mammalian host [79] showed that Tye7, one of the major regulators of carbohydrate metabolism in *C. albicans*, was needed for proliferation in the gut, but not during systemic infections. This observation suggests that regulators of carbohydrate metabolism in *C. albicans* can play a key role in the ability of the organism to exploit different host niches. The role of Tye7 in GI tract colonization is consistent with the currently observed role of Med7, as the Mediator subunit bind directly and regulate the promoter of glycolytic and carbohydrate metabolism genes [80]. The fact that Tye7 is a key regulator of carbon metabolism and loss of Med7 interferes with aspects of carbon metabolism supports a connection between carbon metabolism regulation and colonization.

To more thoroughly investigate the biological functions of the Med7 subunit we monitored various aspects of the *in vitro* phenotype of the disruption strain, and compared this with the location and transcript profiling experiments. This analysis provided a more detailed framework for the observed defect in GI tract colonization. ChIP-Chip and transcription profiling revealed that Med7 appears to be involved in the activation of the entire glycolytic pathway. The Med7 protein was detected at the promoters of all the glycolytic genes when yeast cells were grown on glucose, and the *med7* mutant strain showed a down-regulation of several glycolytic genes under glucose growth conditions. Med7 also regulated metabolic processes linked to the glycolytic pathway such as glycogen metabolism, mannose and fructose metabolism. As well, Med7 strongly bound the genes encoding phosphofructokinase, which catalyzes an irreversible glycolytic-committing reaction. Phenotypically, we found that the *med7* null mutant seems to interfere with aspects of carbon metabolism. In solid media with different carbon sources the *med7ΔΔ* strain grew somewhat more slowly than the WT strain judged by the size of the colony. When liquid media were used with glucose, galactose, mannose, fructose and glycerol carbon sources or without any carbon source, a growth defect for the *med7* strain was observed for non-glucose carbon sources (Figure 4B and S4). While the mutant and WT strains grew with similar kinetics in the SC glucose liquid medium, the mutant strain was slower growing in galactose, fructose, mannose and glycerol media, and failed to grow detectably in the SC medium lacking any carbon supplement.

### Med7 function in *C. albicans*

The mechanism of how the Mediator complex contributes to transcriptional regulation is not yet very well understood, as it involves a complicated network of interactions among the several multi-subunit complexes that make up the transcription machinery. However, Mediator is proposed to act principally during the assembling of the pre-initiation complex [81]–[83]. Evidence from genetic experiments in yeast shows that loss of certain key Mediator subunits disturbs transcription as dramatically as does loss of Pol II [84]; this suggests that the Mediator complex as a whole could be considered a component of the general transcription machinery. However, results of both biochemical and genetic studies suggest that individual Mediator subunits can have remarkably gene-specific or tissue-specific functions [32]. Further, specific subunits are required for Mediator to bind to certain promoter sequences, for example, the Med2 subunit is required at only 5% of all yeast gene promoters and specific subunits are required to interact with certain transcriptional activators or repressors [6].

Our data for *C. albicans* is consistent with reports in model yeasts suggesting that Mediator subunits have both general and specific functions. Overall we characterized 10 Mediator module subunits representing orthologs of genes identified as essential in *S. cerevisiae* that were part of the GRACE collection [60]. Inactivation of the genes encoding Med8 and Med14 generated immediate growth inhibition, while tet-regulated repression of the genes encoding Med4, Med10, Med11, Med17, Med21 and Med22 also blocked growth, but this growth inhibition was not as rapid and extreme. Thus the essential nature of these subunits was conserved from *S. cerevisiae* to *C. albicans*. However, repression of the *C. albicans MED7* ortholog, which represents a subunit showing a high level of structural conservation, had little effect on cell growth in the presence of glucose as a carbon source, although the growing colonies exhibited a wrinkled morphology (Figure 1). This non-essential nature of the Med7 subunit was confirmed by generating a true disruption null mutant that exhibited a slight reduced growth rate when compared to the wild type strain, but was completely viable under normal growth conditions. *med7* mutant was not able to activate properly a subset of genes enriched for activation by specific transcription factors causing an inhibition of filamentous growth in hyphal inducing conditions (Fig. 5A). The specificity of Med7 was observed as well in the pattern of Med7 binding which was altered by transcriptional changes caused by the yeast-to-hyphae switch. In hyphal conditions, Med7 binds several FGR family members (*FGR 6-1, 6-3, 6-10, 6-4, 50, 22, 1, 18* and *34*) as well as cell adhesion factors (*ALS1*, *TDH3* and *DEF1*) and regulators such as *ACE2* and ergosterol genes (*ERG6* and *25*). It also binds genes implicated in the white-opaque switch (*WOR1* and *WOR2*) as well as several biofilm formation regulators (*TRY2, CRZ2, ADH5, TRY6, MET4*). We could not see any direct binding of hyphal specific genes such as *HWP1*, *ESE1*, *MSS11*, *PGA7* or *TEC1* suggesting that Med7 may play an important role in filamentous growth through regulators such as the FGR family. Consistent with the transcriptional down-regulation of several adhesin factor genes including *ALS1*, *ALS3*, the mutants are defective in biofilm formation. Uwamahoro et al., 2012, observed a similar phenotype; they identified a shared function of *ace2* and *med31* mutants that display a cytokinesis defect as well as adherence and biofilm formation phenotypes, suggesting that the transcriptional activator Ace2 could be modulating a number of Med31-dependent effects on gene expression [53].

Previous studies had investigated genome-wide Mediator binding in *S. pombe* [75] and *S. cerevisiae* [73]. In these studies, similar binding patterns were identified regardless of the subunit tagged for the analysis, suggesting the Mediator functions as a coordinated complex. In *S. pombe* Med7 and SRB8-11 subunits gave common binding patterns while in *S. cerevisiae* CycC, Med17, Med19, Med7, Med14, Med3 and Med15 all gave similar patterns. The uniformity in mapping of different Mediator subunits was somewhat surprising with regard to the repressive CDK module [1]. Genomic location analyses in these prior studies of different Mediator subunits indicate a uniformly composed core complex upstream of active genes but unexpectedly also upstream of inactive genes. Because Mediator appears to function as a coordinated complex, analysis of the binding location of any subunit should provide a picture of binding of the general complex assuming characteristics of Mediator are consistent among different organisms. Even though Med7 has a different essentiality in *C. albicans* and *S. cerevisiae*, the genome-wide occupancy of Mediator obtained by analysis of Med7 binding in *C. albicans* in both yeast and hyphal conditions, when compared to the transcriptional activity of individual genes that have been determined previously [72], showed a general distribution as seen for *S. pombe* and *S. cerevisiae*. Our data suggest that there is a common set of 200 genes bound by Mediator in either the yeast or hyphal conditions, and the fraction of Med7 bound genes increases with the higher transcriptional activity observed during hyphal cell growth compared to yeast cell growth. This suggests a positive correlation with transcriptional activity and Mediator binding, and that this binding is condition dependent. We compared the core binding with previous Pol II expression profile in *C. albicans* [72], and we found that Mediator binding was also associated with both active and inactive genes (of the 318 yeast Med7 targets only 139 were active under the conditions assayed).

Overall, Med7 binding in *C. albicans* supports the pattern observed for other Mediator complexes in *S. cerevisiae* and *S. pombe*. First, Med7 binding includes not only the intergenic regions but also coding regions and 3′-ends of some genes. Second, Mediator occupancy correlates with the transcriptional activity changes between the yeast and hyphal growth forms. Third, Med7 binding occurs at both active and inactive genes. We did not detect Med7 binding enrichment at several highly transcribed genes (Table S8); this contrasts with previous implications that Mediator must function as a general transcription regulator for all Pol II transcription [84], [85], but is consistent with the pattern observed in other fungi [75].

The fact that the essential nature of a component of a central metabolic control complex can change from one ascomycete to another is intriguing. Gene essentiality changes are key requirements for organismal evolution. However, it is unclear just how the essentiality of orthologs varies across species. One of the hypotheses to explain the observed variation is that changes in network connections arise through engagement in protein complexes [86]. Genes that are nonessential in yeast have been found to be essential in other species where their network connections are significantly increased. In this context, it is intriguing that the Med7 mediator subunit showed this change in essentiality between *S. cerevisiae* and *C. albicans*. Depletion of Med7 in *S. cerevisiae* cells, by the use of a tetracycline-repressible promoter, resulted in complete arrest of cell division, and the arrested cells were larger than wild type and showed elongated buds [87]. However in *C. albicans* the equivalent reduction of Med7 by a tetracycline-repressible promoter or even by the complete deletion of both alleles of the subunit resulted in viable cells. It is clear that Med7 is still a component of the *C. albicans* Mediator complex, but it will be interesting to see if its connectivity to external proteins such as transcription factors and chromatin elements has changed between the two ascomycetes. Further work will be necessary to fully understand the overall function of Mediator and Mediator subunits, and the genetically and molecularly tractable systems available in the ascomycete yeasts should be important tools in these investigations.

## Materials and Methods

### Yeast strains and growth conditions

Strains used in this study are listed in Table S1. For general propagation and maintenance, the strains were cultured at 30°C in yeast-peptone-dextrose (YPD) medium supplemented with uridine (2% Bacto peptone, 1% yeast extract, 2% dextrose, and 50 µg/ml uridine, with the addition of 2% agar for solid medium). Cell growth, transformation and DNA preparation were carried out using standard yeast procedures.

For gene expression profiling of yeast-form cells, saturated overnight cultures of all strains were diluted to a starting OD^600^ of 0.1 in 50 ml fresh YPD and grown at 30°C to an OD^600^ of 0.8. Hyphae were induced by growing *Candida* cells in YPD plus 10% fetal bovine serum at 37°C to an OD^600^ of 0.8. Cultures were harvested by centrifugation at 3,000× g for 5 minutes, and the pellet rapidly frozen in liquid nitrogen.

For *med7*ΔΔ phenotypic growth on different carbon sources, cells were plated on YP media containing the appropriate carbon source (glucose, galactose, fructose, mannose or glycerol) at 2% and agarose at 2%. The cells were plated as well on YP agar without any carbon source. For liquid assays, cells were grown to log phase in synthetic medium, washed twice with sterile water, and resuspended at an OD^600^ = 0.1 in synthetic media containing the suitable carbon source at 2% or without any sugar source. Cells were grown at 30°C in 96 well plates with shaking using a Tecan –Sunarise plate reader.

### Grace library phenotype screen

All the GRACE mutants were made in the CaSS1 strain background as described [60]. To summarize a suitable parent strain (CaSS1) for GRACE approach was engineered in the *C. albicans* CAI4 strain background by introducing a homozygous his3 auxotrophic deletion mutation and expression of a chimeric tetracycline transactivator protein comprising the *tetR* DNA-binding domain of E. coli fused to the *S. cerevisiae* GAL4 activation domain. Gene essentiality of all Mediator subunit conditional mutants was evaluated using independent methods. Starting from an overnight culture (OD_600_ 3.0), serial dilutions of cells were plated onto both a YPD plate, and a YPD plate containing 100 µg/ml tetracycline and 20 µg/ml doxycycline, and growth was determined after 48 h at 30°C. Independently, gene essentiality was determined by streaking Mediator subunit GRACE cells onto a SC plate containing 1 mg/ml 5-fluororotic acid (5-FOA) to select for ura^−^ cells which have excised the transactivator construct that is normally required for expression of the tetracycline-promoter-regulated target gene [60], [88].

### Med7 knock-out and revertant strains construction

The *C. albicans* strains used in the gene disruption experiments are derivatives of BWP17. The *med7*ΔΔ strain was constructed by standard methods based on PCR and homologous recombination, using *LEU2* and *HIS1* as selective markers as previously described by Gola et al [89]. For reintegration experiments, the *MED7* gene was reintegrated into the null mutant *med7* strain. The wild-type *MED7* gene was amplified from genomic DNA using oligonucleotides REVF1 and REVR1 (Table S1) and Expand high-fidelity polymerase (Roche). The PCR fragment was digested with restriction enzymes *Kpn*I and *Xho*I and cloned in the same sites of the CIp10 vector [90]. Plasmid CIp10-*MED7* was digested with the *Stu*I restriction enzyme and used to transform the *med7* mutant strain. The uridine-positive colonies were analyzed by PCR, and the obtained wild-type fragment confirmed the reintegration of the *MED7* gene.

### Biofilm assay

XTT assays were carried out as previously described [91]. Briefly, overnight YPD cultures were washed twice with PBS and resuspended in RPMI 1640 (GIBCO) supplemented with L-glutamine to OD_600_ = 0.1. Ninety-six well polystyrene plates (Costar) were used and 100 µl of cells were added to each well. The plates were placed at 37°C for 2 h to initiate the biofilm formation, washed 3 times and 100 µl of fresh RPMI media was added. The plate was placed in a rocking incubator at 37°C for 24 hours. The media and any non-adherent cells were removed and the wells were washed three times with PBS. After washing, 100 µl of a freshly prepared XTT-menadione solution (0.5 g/L XTT in PBS and 1 µM menadione in acetone) was added to sample and control wells. The plate was incubated in the dark for 2 hours at 37°C and the colorimetric change resulting from XTT reduction was measured at 490 nm. Six biological replicates done in six replicates were performed.

### Measurement of gastrointestinal colonization

Female Swiss Webster mice (18–20 g) were treated with streptomycin (2 g/L), gentamycin (0.1 g/L) and either tetracycline (2 g/L) or bacitracin (1 g/L) in their drinking water throughout the experiment beginning 4 days prior to inoculation. Mice were inoculated with *C. albicans* by oral gavage (5×10^7^ *C. albicans* cells in 0.1 ml), as described previously [92]. Colonization was monitored by collecting fecal pellets (produced within 10 minutes prior to collection) at various days post-inoculation, homogenizing in PBS, plating homogenates on YPD agar medium supplemented with 50 µg/mL ampicillin and 100 µg/mL streptomycin. CFU/g of material was measured; when no colonies were detected, CFU/g was assumed to be the limit of detection (1 CFU). Mice were sacrificed on day 8 post-inoculation and *C. albicans* concentrations in stomach and cecum contents were measured as above. Homogenates of kidneys, liver, and tongue were measured by plating; no colonies were observed from homogenates of these organs. Composite results from two experiments are shown. Colonization data were analyzed using R [93]. A one-way ANOVA was used to test for differences between colonization conditions. When colonization differed significantly between conditions (p<0.05), post-hoc pairwise t-tests were performed.

### RNA extraction, and microarray experiment

To extract RNA from cells, samples stored at −80°C were placed on ice and RNeasy buffer RLT was added to pellets at a ratio of 10∶1 (vol/vol) buffer/pellet. The pellet was allowed to thaw in the buffer with brief vortexing at high speed. The resuspended pellet was placed back on ice and divided into 1 ml aliquots in 2 ml screw cap microcentrifuge tubes containing 0.6 ml of 3 mm diameter acid-washed glass beads. Samples were homogenized 5 times, 1 minute each, at 4,200 RPM using a Beadbeater. Samples were placed on ice for 1 minute after each homogenization step. After the homogenization the Qiagen RNeasy protocol was followed as recommended. Total RNA samples were eluted in RNAse free H_2_O, and RNA quality and integrity were assessed using an Agilent 2100 bioanalyzer.

cDNA labeling and microarray production were performed as described [94]. Briefly, 20 µg of total RNA was reverse transcribed using 9 ng of oligo(dT)_21_ and 15 ng of random octamers (Invitrogen) in the presence of Cy3 or Cy5-dCTP (Invitrogen) and 400 U of Superscript III reverse transcriptase (Invitrogen).

After cDNA synthesis, template RNA was degraded by adding 2.5 units RNase H (Promega, Madison, WI, USA) and 1 µg RNase A (Pharmacia, Uppsala, Sweden) fol- lowed by incubation for 15 minutes at 37°C. The labeled cDNAs were purified with a QIAquick PCR Purification Kit (Qiagen). Prior to hybridization, Cy3/Cy5-labeled cDNA was quantified using a ND-1000 UV-VIS spectrophotometer (NanoDrop, Wilmington, DE, USA) to confirm dye incorporation. DNA microarrays were processed and analyzed as previously described [95]. The microarray data set has been deposited in GEO, under accession number GSE61519.

### Gene Set Enrichment Analysis (GSEA)

Gene Set Enrichment Analysis (GSEA), was used to determine whether defined lists (or sets) of genes exhibit a statistically significant bias in their distribution within a ranked gene list (see http://www.broadinstitute.org/gsea/index.jsp for details) [62]. This required initially the construction of an extensive gene set and annotation database using publicly available data from CGD, SGD and BioGRID, together with transcription factor binding data from all currently published ChIP-chip experiments, our own TF motif database, lists of modulated genes from both transcriptional profiling experiments, and genetic-association data obtained from SGA screens measuring cell growth.

### Tiling array design

Starting from sequences from the *C. albicans* Genome Assembly 21 [96] and the *MTL* alpha locus [97], we extracted a continuous series of 242,860 60-bp oligonucleotides each overlapping by 1 bp. We then eliminated 2,062 probes containing stretches of 13 or more A or T nucleotides. The remaining 240,798 sequences were then used to produce sense and AS whole genome tiling arrays using the Agilent Technologies eArray service.

### Whole-genome location profiling by ChIP-Chip and data analysis

Med7 (*ORF19.232*) was TAP- tagged *in vivo* with a TAP-*URA3* PCR product as described [71]. Transformants were selected on -ura plates and correct integration of the TAP-tag was checked by PCR and Western blot. Saturated overnight cultures of Med7 tap tagged and WT strains were diluted to a starting OD_600_ of 0.1 in appropriate media. Cells were grown to an OD_600_ of 2 in 40 ml of YPD at 30°C for yeast condition and in YPD plus 10% fetal bovine serum at 37°C for hyphae induced condition. The subsequent steps of DNA cross-linking, DNA shearing, chromatin immuno-precipitation and DNA labeling with Cy dyes were conducted exactly as described by Lavoie *et al.* [71]. Tiling arrays were co-hybridized with tagged immunoprecipitated (Cy5-labeled) and mock immunoprecipitated (untagged SN148 strain; Cy3-labeled) DNA samples. Microarray hybridization, washing and scanning were performed as described above [94]. The significance cut-off was determined using the distribution of log-ratios for each factor. It was set at 2 standard deviations from the mean of log-transformed fold enrichments. Values shown are of two biological replicates derived from independently isolated transformants of tagged and mock constructs. Peak detection was performed using Gaussian edge detection applied to the smoothed probe signal curve as described [71], [98]. The ChIP Chip data set has been deposited in GEO, under accession number GSE61519.

### Immunoprecipitation

*C. albicans* Med7-tap was grown to mid-log phase in YPD media. Cells at a final OD_600_ of 1.0–1.5 were harvested by centrifugation and lysed by bead beating in IP150 buffer (50 mM Tris-HCl (pH 7.4), 150 mM NaCl, 2 mM MgCl_2_, 0.1% Nonidet P-40) supplemented with Complete Mini protease inhibitor mixture tablet (Roche Applied Science) and 1 mM phenylmethylsulfonyl fluoride (PMSF). The lysates were then cleared by centrifugation, and protein concentration was estimated using the Bradford assay. One milligram of total protein was added to 50 ul of anti-Tap IgG sepharose beads (GE) and incubated at 4°C with end-over-end mixing overnight. The next morning, beads were centrifuged at 2000 rpm at 4°C, washed three times with IP150 buffer, boiled with SDS-PAGE loading buffer, and resolved by 4–20% gradient SDS-PAGE. Proteins were transferred onto a nitrocellulose membrane and analyzed by Western blotting using rabbit anti-tap polyclonal antibody (1∶2500) (GenScript).

### TAP purifications and mass spectrometry LC-MS/MS

To assess protein-protein interactions on a large scale we used a Med7 TAP-tag construct. We performed a standard TAP procedure using the TAP-tagged Med7 and the untagged control to identify the specific protein to Med7. Tandem affinity purifications were performed as described http://depts.washington.edu/yeastrc/pages/plasmids.html and then precipitated with trichloroacetic acid (TCA). For mass spectrometry analysis of the TAP purified proteins, 0ne third of the TCA precipitate was loaded on a 10% SDS-PAGE gel. The gel was stained with gel code blue according to manufacturer's instructions (Invitrogen). Entire lanes were cut into 3 bands and subsequently destained, reduced, cysteine-alkylated and in-gel digested with sequencing grade modified trypsin (Promega, Madison, WI) as previously described by Wasiak *et al.* [99]. Peptides were extracted from the gel pieces through multiple incubations in solutions of 1% FA and increasing concentration of Acetonitrile (ACN). The extracts were dried in a Speedvac and resuspended in 60 µl 5% ACN:0.1% FA. Five µl of peptide digest was loaded onto a 15 cm×75 µm i.d PicoFrit column (New Objective, Woburn, MA) packed with Jupiter 5 µm, 300 Å, C18 resin (Phenomemex, Torrance, CA) connected in-line with a Velos LTQ-Orbitrap mass spectrometer (Thermo-Fisher, San Jose, CA). Peptide separation was done using a linear gradient generated by an Easy-LC II Nano-HPLC system (Thermo-Fisher) using a mixture of solvent A (3% ACN:0.1% FA) and solvent B (99.9% ACN:0.1%FA). The gradient started at 1% B, was set to reach 27% B in 26 min, ramped to 52% B in 4 min and 90% B in 2 min then held at 90% for 5 min. The mass spectrometer used was a Velos LTQ-Orbitrap (Thermo-Fisher, San Jose, CA). Raw mass spectrometric data were processed using Proteome Discoverer 1.3. Spectra were searched against a *C. albicans* SC5314 database obtained from www.candidagenome.org containing 6215 protein sequence entries.

## Supporting Information

Figure S1 Invasiveness assay of the Mediator-subunit Grace strains. Cultures of wild type C. *albicans*, and the mediator subunit from Grace collection were grown overnight at 30°C. Strains were serially diluted and grown on plates under standard repressing conditions (YPD+20 µg/ml doxycycline) for 2–3 days at 30°C. Invasive growth was scored by examination of the colony perimeter and by viewing cell retention after washing the colony from the agar surface. The first line show the Mediators strains image before wash and the second line shows the image after wash. All Mediator complex core subunit mutants except the SSN8 showed strongly enhanced invasiveness and produced very dense pseudohyphal cells.
(EPS)

Figure S2 Screen of Mediator subunit mutants in the GRACE collection in hyphal promoting condition. Cultures of wild type C. *albicans*, and the mediator subunit from Grace collection were grown overnight at 30°C. Strains were serially diluted and grown on plates under non-repressing (YPD+10% FBS, Spider or M199) or standard repressing conditions (YPD+10% FBS, Spider or M199+100 µg/ml tetracycline or 20 µg/ml doxycycline) for 2–3 days at 37°C and the resulting colonies photographed.
(TIF)

Figure S3 Generation of *med7* null mutant. *MED7* complete knock out of both alleles of the gene through standard disruption approaches. One allele was replaced with the *LEU2* selection marker and the second allele was replaced by *HIS1*. The total removal of the functional *MED7* gene and its replacement with the selection markers was confirmed by PCR. The wild-type *MED7* gene was amplified from genomic DNA to complement the *med7* mutant. The positive colonies were analyzed by PCR, and the obtained wild-type fragment confirmed the reintegration of the *MED7* gene.
(EPS)

Figure S4 Growth assay in liquid medium with the different carbon sources, as well as with SC not supplemented with any carbon source. The growth curve was generated using a Tecan –Sunrise plate reader during 2 days at 30°C.
(EPS)

Figure S5 5-FOA assay of the Mediator subunit Grace strains. Cultures of wild type C. *albicans*, and the mediator subunit from Grace collection were grown overnight at 30°C. Strains were serially diluted and grown on 5-FOA plates or 3–5 days at 30°C. the cells were re-streaked from 5-FOA plates to YPD and –ura plates to confirm the non-essentiality.
(EPS)

Table S1 Strains and primers used in this study.
(DOCX)

Table S2 Protein-protein interactions of CaMed7 using a TAP purifications and mass spectrometry LC-MS/MS.
(XLSX)

Table S3 Genes regulated in med7 Grace mutant in yeast condition by microarray.
(XLSX)

Table S4 Genes regulated in med7 Grace mutant in hyphae condition by microarray.
(XLSX)

Table S5 GSEA analysis of Med7 Grace mutant in yeast and hyphal condition.
(XLSX)

Table S6 Targets in yeast and hyphae of C. albicans Med7 by full-genome tiling arrays.
(XLSX)

Table S7 Peaks binding in yeast and hyphae of C. albicans Med7 by full-genome tiling arrays.
(XLSX)

Table S8 List of active genes by comparing Med7 and POLII targets.
(XLSX)
