# Supplementary figures and images for: A Functional Portrait of Med7 and the Mediator Complex in Candida albicans
Source: PLoS Genet. 2014 Nov 6;10(11):e1004770. doi: 10.1371/journal.pgen.1004770 (PMC4222720; doi:10.1371/journal.pgen.1004770)

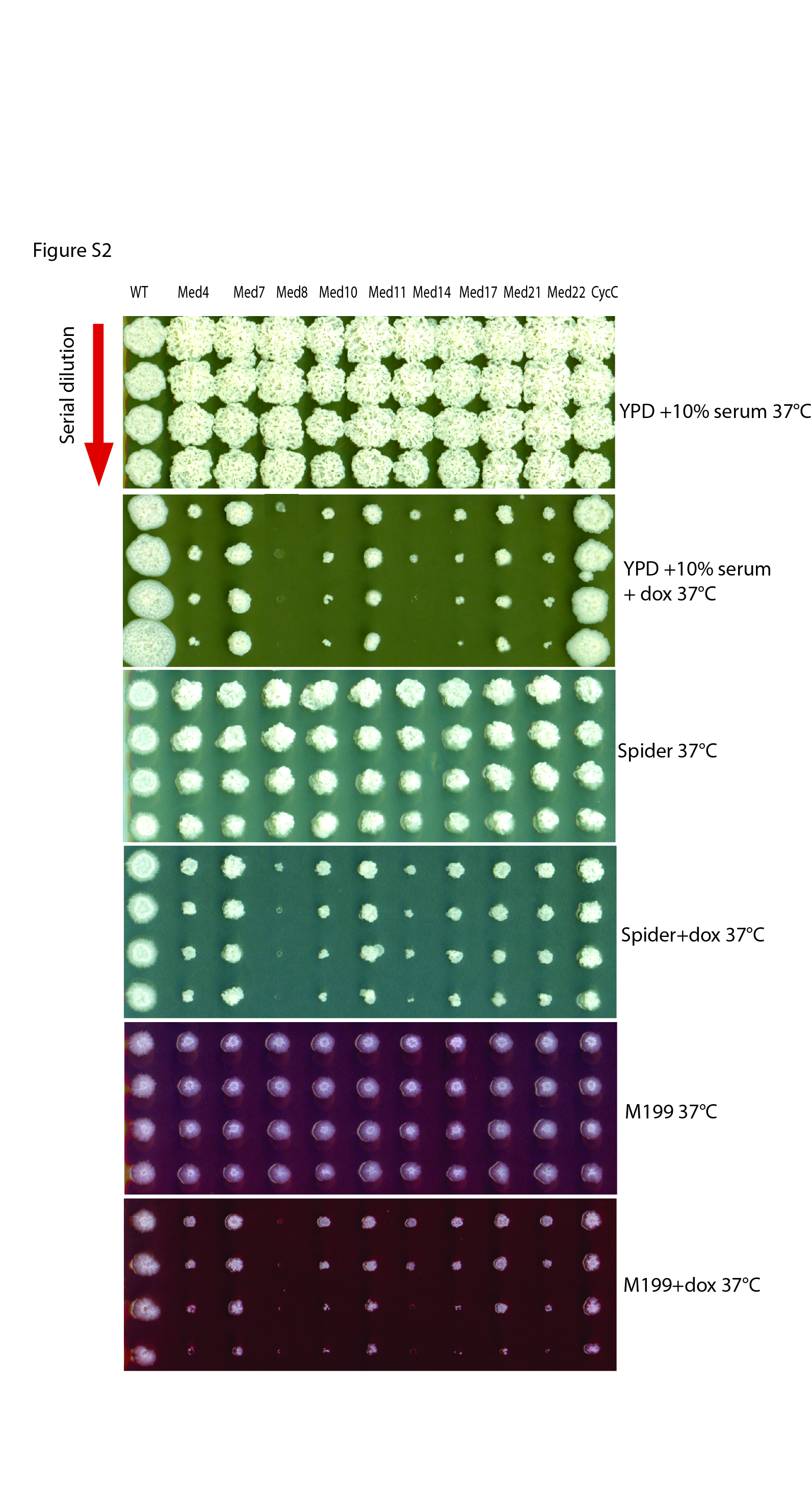

Supplement: Figure S2 — Screen of Mediator subunit mutants in the GRACE collection in hyphal promoting condition. Cultures of wild type C. albicans, and the mediator subunit from Grace collection were grown overnight at 30°C. Strains were serially diluted and grown on plates under non-repressing (YPD+10% FBS, Spider or M199) or standard repressing conditions (YPD+10% FBS, Spider or M199+100 µg/ml tetracycline or 20 µg/ml doxycycline) for 2–3 days at 37°C and the resulting colonies photographed. (TIF) [file pgen.1004770.s002.tif]
