# Supplementary material for: A Functional Portrait of Med7 and the Mediator Complex in Candida albicans
Source: PLoS Genet. 2014 Nov 6;10(11):e1004770. doi: 10.1371/journal.pgen.1004770 (PMC4222720; doi:10.1371/journal.pgen.1004770)
Supplement: Table S1 — Strains and primers used in this study. (DOCX) [file pgen.1004770.s006.docx]

Table S1. 1.*Candida albicans* strains and primers used in this study

| Strain | Parental strain | Genotype or description | Reference |
| --- | --- | --- | --- |
| SC5314  SN148  Cass1  Med4 Grace  Med7 Grace  Med8 Grace  Med10 Grace  Med11 Grace  Med14 Grace  Med17 Grace  Med21 Grace  Med22 Grace  CycC Grace  Med7KO  Med7 Rev  Med7-tap | SN76  CAI4  Cass1  Cass1  Cass1  Cass1  Cass1  Cass1  Cass1  Cass1  Cass1  Cass1  SN148  SN148  SN148 | *Clinical isolate*  *arg4Δ/arg4Δ leu2Δ/leu2Δ his1Δ/his1Δ*  *ura3Δ::imm434/ura3Δ::imm434*  *iro1Δ::imm434/iro1Δ::imm434*  *his3::hisG/his3::hisG leu2::tetRGAL4AD-URA3/LEU2*  *med4::his3::hisG/his3::hisG leu2::tetRGAL4AD-URA3/LEU2*  *med7::his3::hisG/his3::hisG leu2::tetRGAL4AD-URA3/LEU2*  *med8::his3::hisG/his3::hisG leu2::tetRGAL4AD-URA3/LEU2*  *med10::his3::hisG/his3::hisG leu2::tetRGAL4AD-URA3/LEU2*  *med11::his3::hisG/his3::hisG leu2::tetRGAL4AD-URA3/LEU2*  *med14::his3::hisG/his3::hisG leu2::tetRGAL4AD-URA3/LEU2*  *med17::his3::hisG/his3::hisG leu2::tetRGAL4AD-URA3/LEU2*  *med21::his3::hisG/his3::hisG leu2::tetRGAL4AD-URA3/LEU2*  *med22:his3::hisG/his3::hisG leu2::tetRGAL4AD-URA3/LEU2*  *cycc::his3::hisG/his3::hisG leu2::tetRGAL4AD-URA3/LEU2*  *Med7::leu2/med7::his1 ura3/ura3*  *arg4/arg4*  *med7 ΔΔ /*MED7 *ura3/ura3*  *arg4/arg4*  *MED7/MED7-TAP-URA3* | [1]  [2]  [3]  [4]  [4]  [4]  [4]  [4]  [4]  [4]  [4]  [4]  [4]  This study  This study  This study |

| Names | Sequence |
| --- | --- |
| MEd7KOF1 | atgtcaacgaataacaccactacaaacaatgaagatctaatatcctcattatatccaccaccaccgccatactataaattgaagcttcgtacgctgcaggtc |
| MEd7KOR1 | ttgacagtaataatctattaatgatatcttgtttcaattgttccttttcatcaattgaatcaccatcaccaagtcctgtttctgatatcatcgatgaattcgag |
| MEd7ExF1 | ttcctccttcttttcctccaa |
| MEd7ExR1 | ttgctggaggacaaacttca |
| Med7InF1 | cacgtggcaagaaaacaatc |
| Med7InR1 | ccatcacttaccactgttattcg |
| Med7RevF1 | ggtggtggtggtacctgtgtacgcgtaggttgagtg |
| Med7RevR1 | ggtggtgtcgacgctccacgacttatggcatt |
| Med7TAPF1 | aggacttggtgatggtgattcaattgatgaaaaggaacaattgaaacaagatatcattaatagattattactgtcaataGGTCGACGGATCCCCGGGTT |
| Med7TAPR1 | actactactactcctcaacaatcaactcaaagctccacgacttatggcattgaaaacttatctgtatcaggaaatgcatctaaTCGATGAATTCGAGCTCGTT |
| Med7ExTAPF1 | aataccgccaaaacaacctg |
| Med7ExTAPR1 | cgccttcttcgtcttctcaa |
| Tap-Ura3-F | tataggtcttagtgttgactgt |
| Tap-Ura3-R | ttgaaggattaaaacagggagc |
|  |  |

1. Gillum A, Tsay E, Kirsch D (1984) Isolation of the Candida albicans gene for orotidine-5′-phosphate decarboxylase by complementation of S. cerevisiae ura3 and E. coli pyrF mutations - Springer. Molecular and General Genetics MGG.

2. Noble SM, Johnson AD (2005) Strains and Strategies for Large-Scale Gene Deletion Studies of the Diploid Human Fungal Pathogen Candida albicans. Eukaryotic Cell 4: 298–309. doi:10.1128/EC.4.2.298-309.2005.

3. Fonzi WA, Irwin MY (1993) Isogenic strain construction and gene mapping in Candida albicans. Genetics 134: 717–728.

4. Roemer T, Jiang B, Davison J, Ketela T, Veillette K, et al. (2003) Large-scale essential gene identification in Candida albicans and applications to antifungal drug discovery. Mol Microbiol 50: 167–181.
